# Functionality of Food Components and Emerging Technologies

**DOI:** 10.3390/foods10010128

**Published:** 2021-01-08

**Authors:** Charis M. Galanakis

**Affiliations:** 1 Research & Innovation Department, Galanakis Laboratories, P.C. 73131 Chania, Greece; cgalanakis@chemlab.gr; 2 Food Waste Recovery Group, ISEKI Food Association, P.C. 1190 Vienna, Austria

**Keywords:** bioavailability, bioactivity, bioaccessibility, non-thermal, functional foods, nutraceuticals, food additives

## Abstract

This review article introduces nutrition and functional food ingredients, explaining the widely cited terms of bioactivity, bioaccessibility, and bioavailability. The factors affecting these critical properties of food components are analyzed together with their interaction and preservation during processing. Ultimately, the effect of emerging (non-thermal) technologies on different food components (proteins, carbohydrates, lipids, minerals, vitamins, polyphenols, glucosinolates, polyphenols, aroma compounds, and enzymes) is discussed in spite of preserving their functional properties. Non-thermal technologies can maintain the bioavailability of food components, improve their functional and technological properties, and increase the recovery yields from agricultural products. However, the optimization of operational parameters is vital to avoid degradation of macromolecules and the oxidation of labile compounds.

## Highlights

The review introduces nutrition and functional food ingredients, explaining the terms of bioactivity, bioaccessibility, and bioavailability;
The effect of emerging technologies on different food components is discussed in spite of preserving their functional properties;
Non-thermal technologies can maintain the bioavailability of food components and improve their functional properties;
They increase the recovery yields of food components from agricultural products
Optimization of operational parameters is important vital to avoid degradation and oxidation of labile compounds.

## 1. Introduction

Processing of foods has raised public interest and researchers’ attention due to the numerous references of international organizations (e.g., the Food and Agriculture Organization and the World Health Organization) about chronic diseases induced by lifestyle habits along with unhealthy diets [1,2]. For instance, the so-called “metabolic syndrome” and obesity has been correlated with the lack of exercise and reduced activity, which are among the major problems affecting modern societies [3]. The eating patterns of a previous couple of decades based on the increased consumption of fast and highly processed foods may affect individuals’ health status [4].

On the other hand, consumers continuously become more aware that proper food is directly connected to their physical wellbeing and can prevent nutrition-related diseases [5,6,7]. Morever, following the catastrophic effects of the COVID-19 pandemic, immunity is among people’s highest priority in the era, and subsequently, consumers are eager to turn their diets to healthier choices [8,9]. Today, the majority (up to 72% in a European study of FMCC Gurus, [10]) of shoppers are seeking nutritious foods that provide not only a balanced calorific content but also functions that promote health [11,12]. While the primary concern of the food companies is to provide safe products [13], the caloric, nutritional and functional composition of foods is also raising interest in the food industry as a result of consumers’ concerns. Besides, governments and authorities worldwide endorse this trend by supporting the development of convenient, qualitative, and healthy foods through multidisciplinary research programs [2].

Throughout history, bioactive compounds have been utilized for treatment purposes due to their therapeutic effects. The new trend is to recover bioactive compounds that physiological effects on living organisms [14]. The development of an efficient and green recovery is, thus, among the current challenges in the food industry. Likewise, researchers are not only seeking for new bioactivity of food components but also putting effort into killing two birds with one stone: reutilizing food processing by-products (thus protecting the environment from undesirable waste) and recovering respective functional compounds that have health benefits and could eventually replace synthetic additives [15,16,17].

On the other hand, the development of foods fortified with bioactive components is continuously increasing in the battle with nutrient deficiency. This trend has led researchers to seek new ways that bioactive compounds affect human health. Bioactive compounds could be recovered from food processing by-products, medicinal plants, and other natural resources. Even though many merchandized food products are marketed as “functional,” the bioavailability of respective bioactives and health claims are still not thoroughly explored. This review article aims to explain the widely cited terms of bioactivity, bioaccessibility, and bioavailability, before discussing the interrelation of these properties with food compounds and the emerging (non-thermal) technologies applied nowadays the food industry.

## 2. Functional Foods

Foods contain ingredients and bioactive compounds such as lipids, peptides, and antioxidants important for human nutrition. The idea of producing foods with boosted functionalities of inherent components has led the food industry to a surge of research activities and the development of “functional foods” [2]. This term was generated by Japanese researchers who were the first to define that food could have a more advanced role for the human body than just nutrients’ supply and gastronomic pleasure. Subsequently, Japan became the first country (European countries and the United States later on) to adapt the relevant FOSHU-legislation (Foods of Specified Health Use) and merchandized numerous products in the market [18]. However, which is the “functional food”? Initially, it should be a food (not a pill or a drug) that induces beneficial effects on the human body after its consumption in regular amounts within a given diet. Whole vegetables and fruits comprise the simplest and most representative example of this kind of food because they contain high amounts of bioactives that protect human’s cells against oxidative damage, lowering the risk of developing chronic diseases and cancers [2].

However, the United States and European countries have different definitions for relevant products, and the lack of consensus leads to confusion among consumers, researchers, and professionals. For instance, the American Dietetic Association (ADA) refers “all food can be incorporated into a healthful eating plan—the key being moderation and variety.” It classifies all food as functional at some physiological level, pointing out that “the term functional food should not be used to imply that there are good and bad foods” [19]. In the United States, the term “nutraceutical” is also prevalent. It reflects any material (e.g., food or a bioactive part of it) that supports the human body for the prevention and treatment of chronic diseases or provides any health benefits to it [18]. Although nutraceuticals render demonstrated physiological benefits, they are products (not foods) derived in capsules, liquids, or pills, and lately have been grouped with natural and health products [4]. The U.S. regulatory framework makes a firm separation between products intended to be used as drugs and those intended for use as food. There are two subcategories of food in the U.S. regulatory framework (medical food and food for special dietary use) intended to meet special dietary needs of people with existing disease [20]. In the European Union, harmonization was achieved in 2006 with Regulation (EC) No. 1924/2006 on nutrition and health claims made on foods, which requires authorization of all health claims before entering the market [21].

Nevertheless, consumers show a preference for natural products and ingredients that exist inherently in foods instead of receiving compounds that are chemically synthesized as they want to obtain the desired health benefits within their diet and not as a medicinal pill [6]. Besides, consumers believe that minimally processed foods have advanced health benefits than the processed ones. However, this assumption may not be valid for target compounds such as tomato lycopene [4]. The increase of functional foods market can also be explained by other factors, e.g., the improved life quality of older people, the steady increase of life expectancy, and the healthcare cost [21] that leads policymakers to take action upon promoting functional foods [4].

Functional food ingredients have been investigated as additives to fortify foods with enhanced technological properties and develop food products with health claims [15]. During the pandemic, bioactive components have also been suggested to support the immune system against COVID-19 disease [8]. Different epidemiological studies have denoted that the consumption of target nutrients has been associated with a reduced risk of diabetes, stroke, coronary heart, and other diseases. This conclusion has been observed for both nutritional micro-and macro-molecules.

For example, macromolecules (e.g., proteins) find applications in foods and beverages as stabilizers, in confectionery as flavor enhancers, and dairy products as fat replacers [22,23,24,25]. Similarly, pectin, β-glucan, and other soluble dietary fiber can be used as stabilizers in emulsions and fat replacers in food due to their viscoelastic properties. Besides, they can improve products’ shelf-life and reduce lipids’ level of blood [26,27]. Glucosinolates and isothiocyanates (the derived forms) have been linked with the reduction of risk of degenerative diseases (e.g., alimentary tract’s and cancers’ diseases). At the same time, they could be used as flavorings (e.g., in mustards) and antimicrobials [28]. Tocopherols, polyphenols, ascorbic acid, carotenoids, and other antioxidants have been correlated with the reduction of oxidative stress, retardation of aging, and the prevention of arteriosclerosis. Natural antioxidants have also been proposed as UV booster agents in sunscreens and preservatives to fortify vegetable oils, meats, and other foods [15,27,28,29,30,31]. Flavonoids like epigallocatechin gallate and kaempferol, and polyphenolic acids such as protocatechuic have been suggested as potential inhibitors of the main SARS-CoV-2 protease in molecular docking studies [7].

Following this trend, innovative products made of fruits, milk, or vegetables with higher bioactive ingredient contents and increased antioxidant capacity have been introduced in global markets over the last years [32,33]. The food industry claims more recognition of immune-boosting ingredients [34]. For example, a couple of companies have merchandised chocolate balls containing β-glucan from mushrooms, promoting positive health benefits and improving children’s immunity during the post-lockdown period [35]. The development of functional foods involves not only nutritional but also sensory aspects and consumer preferences upon texture, mouthfeel, color, and taste of foods [2]. Looking at food manufacturers who face different technical challenges during the fortification of functional foods, the preferred formulations include easy-make smoothies and beverages. For soft solid products like yogurts, structural properties and stability are very important for both consumers and manufacturers [36]. However, for all kinds of food formulations, the bioavailability of nutrients is the essential characteristic. Thereby, food processing should be precisely designed to maintain not only the organoleptic character and the quality of the food but also the functionality of bioactive ingredients during storage and consumption [2].

## 3. Functionality of Food Components

**Figure 1:**
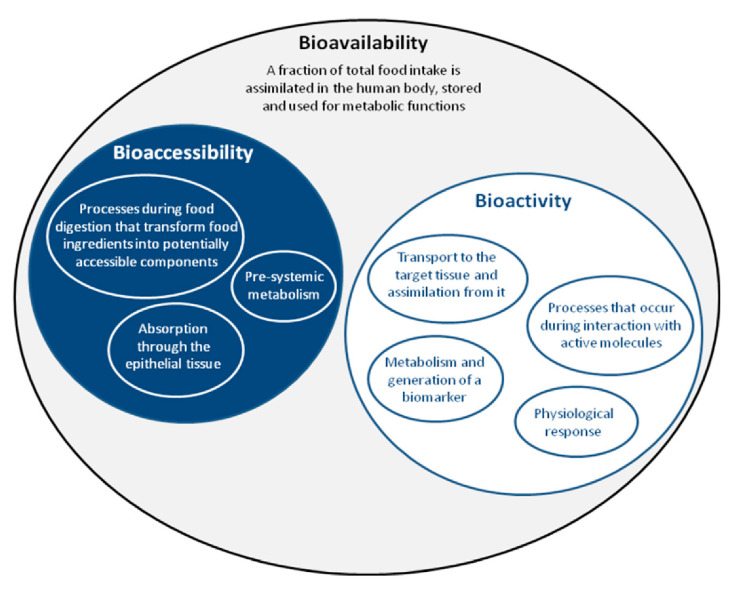
Bioavailability is defined as the synthesis of bioaccessibility and bioactivity, followed by different physicochemical processes. Adapted from Fernández-García et al. [38].

The effectiveness of food bioactives depends on different parameters such as metabolomics, nutrigenomics, bioavailability, and their stability within the food matrix. For example, the delivery of food components via the oral route is restricted by extreme pH, the mucus layer, the epithelium, and the gastrointestinal enzymes. Functional foods are generated after manufacturing traditional or innovative food products with compounds that change their properties (e.g., by changing structure, binding, or interface) and provide health benefits to them. Foods’ preparation requires evaluating different parameters including the selection of relevant sources, the identification of target compounds, the choice of recovery approaches and technologies, conducting toxicological tests when needed, and ultimately performing bioaccessibility, bioactivity, and stability assessments [37]. Therefore, it is necessary to define the relevant terms bioaccessibility, bioactivity, and bioavailability that are typically intrinsically used to express similar and relevant functions (Figure 1).

### 3.1. Bioavailability

Depending on the research area, the bioavailability reflects different meanings. For instance, in pharmacology, bioavailability demonstrates the extent and rate to which molecules (with therapeutic properties) are absorbed in the active site of the drug and become available. In the food field, it reflects the portion of ingested food compounds that reach the systemic circulation and are utilized [39]. When different food matrixes and ingredients are chewed up in the mouth and come in contact with the digestive tract, numerous processes co-occur, affecting bioactive components’ bioavailability. For example, a food ingredient such as fat enhances certain antioxidants’ bioavailability (e.g., quercetin) in meals [40]. Finally, in nutrition, bioavailability refers to the nutrients’ portion stored in the human body or becomes available in physiological actions [41]. Since not all the quantities of bioactive compounds are utilized efficiently by the organism [42], bioavailability is the key to nutritional effectiveness, including all the relevant events such as bioactivity, metabolism, gastrointestinal (GI) digestion, tissue distribution, and absorption. The estimation of bioavailability is conducted with in vivo assays [43].

Bioavailability is the most important property for all the definitions and approaches of functional foods, such as when health or nutritional claim is declared. Initially, the compound or food ingredient bearing the active property should be adequately digested. Once being absorbed and assimilated, the beneficial function for the body takes place [38]. Bioavailability is affected by different factors such as consumer characteristics (e.g., age, gender, condition, and nutrients concentration in the blood), nutrients’ chemical form, nature of the food matrix, etc. The bioavailability of larger molecules (e.g., fats, polysaccharides, and proteins) is typically up to 90% or more of the ingested amount [44]. Variability in the bioavailability of food components may be observed by the lack of homogeneity of food matrix, different food processing or activity of the enzymes [45]. Once foods (or drinks) are consumed, the first step for making a nutrient bioavailable is bioaccessibility [44].

### 3.2. Bioaccessibility

It is a part of bioavailability and is defined as the compound’s amount released from the food matrix in the gastrointestinal (GI) tract, entering the bloodstream and becoming ready for absorption [46]. It reflects that food components should first be released from the food matrix and digested before becoming bioavailable [38]. Therefore, it includes the following events: (i) the transformations of foods during digestion into fractions available for assimilation; (ii) the absorption into intestinal epithelium cells, and (iii) hepatic, intestinal, and pre-systemic metabolism (Figure 1). Nutrients are absorbed up to one percentage that depends on the activity of digestive enzymes in the pancreatic juice and intestine, the decomposition of the food matrix and nutrients’ release, intestinal mucosa’s uptake, the transfer across the gut wall to the lymphatic circulation, the systemic distribution, and deposition, as well as the metabolic and functional use [47]. Other factors that affect bioaccessibility include the antagonism, interactions, and synergies of the different food compounds that involve the materials to be digested and subsequently being available to the body [38].

Before reaching any claim for potential health benefits of food compounds, we have to analyze if the digestion affects the stability and function of bioactive compounds [43]. Nevertheless, the positive effects of calcium-binding of bile salts and other unabsorbed nutrients are not included in such absorption-based definitions [43]. The determination of bioaccessibility is typically conducted by simulating small intestinal digestion with in vitro procedures. In some cases, Caco-2 cell uptake is also evaluated for the overall estimation of bioaccessibility [48]. Bioaccessibility struggles to become an essential parameter for functional foods because the standard experimental models did not distinguish bioaccessibility efficiency and assimilation with bioavailability.

### 3.3. Bioactivity

Bioactivity reflects what happens after the assimilation through the epithelium [43]. It is a target effect upon exposure to a substance. It includes the transportation of bioactive components up to a specific tissue, generating biomarkers and corresponding physiological responses (e.g., anti-inflammatory, antimicrobial, antioxidant, etc.), biotransformation characteristics, interaction with biomolecules, and metabolism (Figure 1). There is plenty of scientific evidence that food compounds exhibit bioactive properties such as blood pressure-lowering, anti-cancer, neuroprotective, antioxidant, antimicrobial, and anti-inflammatory [49,50], contributing to the proper function of the human immune system and wellbeing [51]. Digestibility concerns, particularly the moiety of food ingredients that is bread down into eventually accessible components through different chemical and physical events occurring in the lumen [43]. The assimilation reflects transepithelial absorption’ mechanisms that lead to the uptake of bioaccessible compounds through the epithelium [52]. The bioactivity term applies only to the non-digestible dietary fiber that is well known to provide beneficial health properties to the body, although not being absorbed [53].

The scientific background that accompanies health claims of foods (e.g., reduced disease risk) is based on bioactivities. The typical experimental trials applied to estimate bioactivity are adjusted separately for each health benefit [54]. There are different determinations for bioactivity (in vitro, in vivo, and ex vivo), and all of them are based on processes that occur during the interaction of bioactive compounds with different molecules [43]. The latest event accelerates a signal or produces a response or a metabolite that keeps amplifying until the health benefit (systemic physiologic response) is generated.

Nevertheless, ethical and practical issues are raised when determining compounds’ bioactivity on the organs’ specific sites. In such cases, the portion of the oral dose of an active compound (derived from a thorough preparation) that reaches the system circulation defines the bioavailability [55]. Subsequently, bioavailability is estimated by conducting in vivo trials as the area under the compound’s plasma-concentration obtained after administration of a chronic or acute dose [56]. The nutritional claims of foods (e.g., compared with the health claims of foods) are estimated by bioaccessibility assays without the necessity of conducting bioactivity tests [38].

### 3.4. Bioactive Compounds, Functionalization and Interactions

Whole grains, fruits, and vegetables are rich in food bioactives [57]. The latest comprises different classes as tocopherols, organosulfur compounds, carotenoids, glucosinolates, polyphenols, betalains, and phytosterols. These compounds are very heterogeneous despite their structures, distribution in nature (e.g., ubiquitous or found in individual plants) and characteristics (e.g., lipophilic or hydrophilic), but also about their active site, concentrations in the human body and foods, efficiently against oxidative species, and biological action [43,58]. The interactions between the different biomolecules and the source of origin are among the main factors affecting the bioavailability of food components [36]. For example, carbohydrates (conjugated or free) are found mainly in plant materials. In contrast, macromolecules like proteins, dietary fiber, and lipids are typically bonded in clusters with antioxidants within the fruit matrix [50].

Bioactives promote health by modulating metabolic processes and exhibiting beneficial effects, e.g., antioxidant activities, inhibition receptor activities, induction of enzymes, and gene expression [59]. Nevertheless, when investigating the effect of bioactives in human health, bioaccessibility and bioactivity are not always adequately known [43]. In particular, the most abundant bioactives of vegetables, fruits, and other foods don’t need to be leading to the highest concentrations of active metabolites in tissues [50]. Besides, food manufacturers often claim that some of their food products have a beneficial attribute, but these claims need to have real evidence. For instance, many scientific reports emphasize that most of the beneficial effects regarding the bioactivity of food components have been demonstrated in laboratory tests and not with in vivo experiments that are often difficult to assemble. This observation is valid as a single compound, and single effect relation cannot be explored due to many possible interactions of bioactive with the gut microbiota [60]. Besides, in contrast to the systematic in vivo studies regarding bioactives in the pharmaceutical industry (where each health benefit and the side effect is monitored), there is a lack of elaborated investigations about the role of bioactive compounds in the food industry. An exception is the flavonoid database developed by the USDA [61]. Recently, some evidence from a randomized population and clinical trials has proposed that vitamin D levels may be linked to COVID-19 transmission and severity [7].

During the fortification of foods with bioactive compounds, the food industry focuses mainly on preserving the product’s sensory characteristics (e.g., taste, flavor, odor, and color) [62]. However, it is also essential to retain the functionality of these compounds. The first step is finding out the correct amount of the bioactive compound added to the food to affect health (e.g., prevent certain diseases) during consumption. The next one is to explore the systems required not only to protect bioactive compounds in the food matrix but also protect them during consummation [63]. The design of bioactives’ delivery systems in the food industry is similar to the one in pharmaceuticals, aiming to increase their solubility to reach their target absorption area via systemic circulation, control the release rate, and mask any undesirable organoleptic properties. The stability of bioactive compounds during production, processing, encapsulation, and shelf-life against biochemical, chemical, and physical degradation is also crucial [64]. The solubility and bioavailability of food compounds can be increased by reducing particle size and by using proper delivery formulations that protect bioactives from enzymatic and chemical degradation up to reach the desired place in the GI. These formulations typically include the preparation of emulsions with the help of proteins, biopolymers, lipids, or hydrocolloids (gels with implanted droplets and covalently bound polysaccharides) [65]. Low molecular weights surfactants in micro, nano, and micellar structures, liposomes derived from eggs or sunflower, PEGylated liposomes, organogels, or saponin derived from tea have also been suggested as proper delivery systems [66]. Other examples include resistant starch as a carrier for oral colon-targeting drug matrix system [67], soy protein cold-set hydrogels [68], calcium alginate microparticles for oral administration [69], and innovative covalently conjugated biopolymers [70] among others.

## 4. Food Processing and Emerging Technologies

The sensory and physical properties of foods depend on their ingredients, and food processing directly impacts them. Specifically, processing technologies can affect the functional properties (e.g., bioactivity and bioaccessibility) of food components and, subsequently, their potential health benefits. As extensive as the food processing and as prolonged are storage and transportation, as high as the loss of food bioactives. It is thus essential to control the disintegration degree of the fruit and plant tissues as it affects components’ functionality and foods’ deterioration and quality. Following the increasing demand for nutraceuticals and functional foods, researchers and the food industries have accelerated their efforts in developing processing technologies targeting preserving products’ qualitative, active, and nutritional characteristics [44].

Mechanical pressing, wet milling, and membrane filtration (e.g., microfiltration) are three typical processing techniques in the food industry for the removal of water, tissue softening, and the removal of solids and fats, respectively. Standard technologies like ultrafiltration, isoelectric precipitation and alcohol precipitation can separate macromolecular ingredients such as pectin and proteins from juices and wines. At the same time, acid hydrolysis [71] is used as a pretreatment step, and solvent extraction is applied to recover antioxidants and glucosinolates [27,72,73,74,75]. These technologies, together with thermal product formation (e.g., spray drying) and pasteurization, are typically implemented during the preparation of fortified and functional foods.

All these conventional techniques are well established in the food industry, although facing numerous problems that, in many cases, restrict their applications and cause practical problems. For example, organic solvents are usually not green and food-grade, raising consumers’ awareness about their safe food chain implementation. Thermal sterilization and traditional cooking methods (e.g., blanching, boiling, and frying) have been widely applied to soften plant tissues in the food industry and subsequently for food safety purposes via inactivating bacteria, yeasts, and molds that ferment and reduce the shelf-life of foods. Likewise, many of the detrimental changes during the storage of foods are accelerated by different enzymes (namely pectin methylesterase, polyphenol oxidase, and peroxidize) that are also inactivated by heat stresses. Nevertheless, thermal treatment of food causes degradation of food components. These oxidation and isomerization reactions lead to reduce the loss of quality and finally reduced (instead of prolonged) shelf-life of foods. Besides, over-heating of the food matrix often accelerates undesired Maillard reactions that diminish the sensory properties of foods [15,76]. The bioaccessibility, functionality, and stability of food bioactives are affected, too [77]. Other conventional non-thermal processes (e.g., nanofiltration, freeze-drying) that require high pressures have increased energy demands and operational cost. Besides, the encapsulation of food bioactives with conventional techniques may lead to hazardous products.

These restrictions and disadvantages have led food processors to seek new processing technologies that allow preserving the functionality of bioactive compounds and qualitative characteristics of foods. On the other hand, consumers demand more tailor-made products with extended shelf-life and the sense of “fresh” and minimal preparation time and effort during cooking. This combination of food processors approaches and consumer demands has forced innovators to develop milder processes based on non-thermal concepts or volumetric heating forms where thermal energy reaches directly the core (not only on the surface) of the food matrix, leading to accelerated heat and mass transfer [78]. These technologies (often referred to as “emerging”) offer minimal treatment, preservation of foods’ sensorial characteristics with subsequent protection of food bioactives and their functionality within prolonged storage periods. Emerging technologies have found numerous applications in research and the food industry, promising reduced heating and residence time, improved energetic yield and product’s quality, control of Maillard reactions, and protection from environmental stresses [15,29,73,79,80,81,82].

The most common emerging technologies in the food industry are high-hydrostatic pressure, ultrasound-assisted and microwave-assisted extraction, pulsed electric field, radio-frequency drying, high voltage electrical discharge, and supercritical fluids. Other emerging technologies under development from food manufacturers include cold plasma treatment, electro-osmotic dewatering, and nanoencapsulation [79]. Emerging technologies are not based on high temperature but on the generation of heat through internal energy transmission (e.g., resistive and adiabatic during with pulsed electric fields and high hydrostatic pressure, respectively) that minimizes deterioration of sensory, nutritional, and functional food characteristics.

For instance, high-pressure processing (HPP, typically applied up to 200 MPa) is often used to inactivate microorganisms by damaging their membranes or modifying whey proteins hydrophobicity. It increases the permeability of plant cells, thus accelerating the mass transfer rate of bioactives and allowing diffusion in phase transition [83]. This technology offers faster solubilization of food components without affecting the color, flavor, and texture of foods [77]. Supercritical carbon dioxide extraction is another technology involving the application of gas above its critical pressure and pressure. Although being a fast and selective process, it has been mainly proposed to recover high added-value compounds (e.g., fragrances from natural sources, extraction of target flavonoids, etc.) due to its high operational cost [15,84].

Ultrasound is another non-thermal technology that is known to accelerate mass transfer by generating cavitations in the food matrixes when applied in high-intensity (>10 W/cm^2^, 100 kHz) mode [85]. On the other hand, low-intensity (<1 W/cm^2^, 100 kHz) modes are utilized in the food industry as a tool to monitor a process and obtain information about different physicochemical properties such as milk coagulation, air bubbles in aerated foods, cracks in cheese, quality of eggs, the ratio of fat in meats, the texture of biscuits, wine fermentation control, or fruit, dough and fruit characterization [86,87,88,89]. Ultrasounds are very efficient against pathogens contaminating fruit juices as they have been referred leading to a 5-log reduction (U.S. Food and Drug Administration requirements) of *Escherichia coli* in guava and orange juices [90]. However, the in vitro bioaccessibility of lycopene is known to be reduced during ultrasound processing of tomato pulp as a result of pectin release after a partial de-esterification [91]. Ultrasounds have also found application in modern encapsulation (e.g., 10–100 nm multiphase colloidal nanoemulsions preparation using ultrasounds) aiming at delivering nutrients and bioactives at nano-size (compared to classical roto-stator dispersions) that allows higher bioavailability, physical stability, and energy, as well as controlled release to the target tissues of the human body [92,93,94,95].

Among the modern drying approaches, the conjunction with radio-frequency has been suggested as an alternative technique. With radio-frequency drying, water evaporates below 80 °C at a shorter processing time, and the food matrix is heated uniformly. However, these techniques have low energy efficiency [78]. Electro-osmotic dewatering is a relevant approach that reduces energy consumption by two-thirds compared to traditional hot air drying. It is working by applying mechanical pressure with the simultaneous formation of double layers formed at colloidal particles’ interface with water suspensions [44,79].

The accelerated mass transfer has also been proposed using pulsed electric fields that can disrupt the integrity of cell membranes, soften the texture and electroporation of plant tissues, but also to enhance the recovery of valuable compounds from different vegetables and fruits [83,96]. Pulsed electric fields have been shown to preserve nutrients of milk, eggs, and juices and improve the encapsulation of bioactive compounds by intensifying fluid bed agglomeration of instant soy protein isolate [97].

Another relevant technology is high voltage electric discharge, which has been assayed to recover high added-value compounds from grape seeds [98]. In this application, the extracts are placed in a chamber between two electrodes that disrupt food ingredients by providing short pulses (e.g., 40–60 kV/cm, 2–5 μs) [79]. Similarly, in ohmic heating, the food is placed between two electrodes before an alternating electric current is passed through the circuit. The electrical resistance generates a very rapid and uniform heat generation in food. This technology is comparable to microwave heating but without the intermediate step that converts electricity into microwaves [99]. Ohmic heating has been applied for the pasteurization and sterilization of shear-sensitive, liquid, and solid foods such as poultry, fruits, fish, vegetables, and ready-to-serve meals as for foods thawing [100]. Low-temperature (or so-called “cold”) plasmas are quasineutral particle systems of semi-gas and semi-fluid mixtures of highly active moieties. Cold plasma is generated by electric discharges implemented at different levels of pressure in high-moisture foods, as a vacuum is boosting liquid conversion to gas. This innovative technology has been tested for microbial de-activation in foods [79,101].

Emerging technologies have, in some cases, different drawbacks regarding food safety, efficiency, energy, and operational cost. For instance, cold plasma and ultrasound are advanced oxidation processes that may cause detrimental effects to the bioactive lipids instead of preserving their activity. Besides, the ability of nanoemulsions or cold plasma to insert cells membrane have unknown impacts on biological matrices [79]. It is thus essential to investigate further these effects. On the other hand, food processing at low temperatures allows the gentle treatment of crucial vitamins and the preservation of nutrients and flavors. The interactions between different food bioactives are also others when applying emerging technologies. For example, antioxidants’ interaction with macromolecules may be further in low-processed in highly-processed foods, e.g., binding to dietary fiber instead of proteins [102]. Nevertheless, following market demand for tailored-made products and consumer preferences for milder processes, the adaption of non-thermal technologies accelerates, leading to a new state of the art in functional foods development that needs further exploitation.

## 5. The Effect of Emerging Technologies on Food Components

During food processing, the nature of the implemented technologies affects the effectiveness and the content of bioactives, e.g., as foods are more processed, stored, and transported, as higher is the diminution of the functional properties of contained bioactives. The application of emerging technologies and other issues in the food industry (e.g., the urgent need for innovations and sustainability, food waste recovery, etc.) have brought new data and state of the art in the objectives related to bioavailability. This trend changed the way that bioactives are incorporated inside foods and consumed. Table 1 presents in brief the effects of emerging technologies on different food components.

### 5.1. Proteins

Food proteins are highly polymeric and complex molecules that are comprised of 20 different building units (the amino acids) that are linked between them substituted amide bonds. They can be found in various foods, mainly in meat and dairy products, fish, eggs, legumes, cereals, and oilseeds [99]. Food proteins derived from animals are more wholly digested compared to those derived from plant sources. The quality and functionality of proteins depend on the sequence, composition, and digestibility of essential amino acids, physical and chemical properties like shape, size, distribution of charges, secondary, tertiary, and quaternary structures, molecular flexibility, and hydrophobicity/hydrophilicity ratio, hydrodynamic properties such as texturization, gelation, and thickening, and finally topo-graphical and physicochemical properties such as dispersibility, emulsification, foaming, wettability, solubility, and fat and flavor binding [176].

Several studies have demonstrated that food processing with emerging technologies affects food proteins’ content and functionality [177]. For instance, ohmic heating has been shown to reduce the degradation of seafood significantly and surimi (obtained from squid mantle muscle) proteins (actin and myosin), forming more rigid and elastic gels [103,104,105]. In particular, a very rapid ohmic heating of the paste (from 0 to 90 °C in less than a minute) inactivated metalloproteinase, which is responsible for the degradation of myosin during conventional heating.

On the other hand, the implementation of HPP accelerates the unfolding of the secondary, tertiary, and quaternary food proteins structures (the primary structure is not affected) by dissociating the non-covalent, ionic, hydrophobic, and hydrogen bonds [178]. To this line, it can be used not only for microbial inactivation but also for proteins’ stabilization, e.g., boosting enzymatic proteolysis to hydrolysates with lower residual antigenicity [179]. Other applications include improving surimi’s emulsifying properties and gelling capacity of gel, fish, meat, egg, and soy albumin proteins [99,106,107,108]. Besides, the application of 103 MPa in meat proteins for 2 min resulted in improved digestibility without affecting the biological value or protein efficiency ratio [109]. Similar conclusions (negligible loss of beneficial substances, higher glucose retardation index, and water retention) were obtained by treating tomatoes, carrots, and broccoli with HPP (600 MPa) [110]. PEF treatment also has a minimal effect on food proteins. For example, Jeantet et al. [111] and Fernandez-Diaz et al. [112] did not mention any permanent conformation, denaturation, and precipitation of ovalbumin during the application PEF (20–35 and 31.5 kV/cm strength, respectively) in diafiltrated egg white samples. Nevertheless, the application of prolonged duration pulses width and high-intensity electric fields (45–55 kV/cm) can result in partial modification of egg white protein and milk ß-lactoglobulin [113,114].

The structure-function relationship in proteins may be modified during sonication due to the acceleration or retardation of enzymatic (due to the action of β-glucosidase, invertase, and glycosidase) and chemical (e.g., oxidation, phosphorylation, glycosylation, methylation, acylation, and hydroxylation) reactions [99]. For example, Guzey [115] investigated the impact of high-intensity ultrasound on the functionality and structure of bovine serum albumin, finding out that it increases its charge, hydrophobicity, surface activity, and subsequently emulsifying properties. Likewise, high-power ultrasound enhances protein solubility, foaming capacity, and flexibility (e.g., in whey proteins [116]) by modifying structure and conformation by opening hydrophilic parts of amino acids toward water [180]. Nevertheless, some adverse effects when using ultrasound without testing the right frequency and power for a given treatment time may infer protein denaturation [181,182]. The latest can also be implied by applying γ-irradiation, which breaks down protein into smaller molecules that, upon digestion, yield the same amino acids as the original proteins [117,118]. Other studies have shown that irradiation also affects viscosity, solubility, and stability of gluten, glycerol, and caseinates, as well as fish muscle, soy, and whey proteins [119,120,121].

### 5.2. Carbohydrates

Carbohydrates comprise a group of chemically defined substances that exhibit different physiological and physical properties and health benefits. The primary classification of carbohydrates is based on structural features such as the polymerization degree and linkage type (α or β) [183], including water-soluble monosaccharides (e.g., D-glucose, D-mannose, D-galactose, L-arabinose L-xylose, and D-fructose) and disaccharides (e.g., lactose and sucrose), as well as those derived from β-glucans, dextrins, galactooligosaccharides, xylan oligosaccharides, oligofructans, and short-chain pectin. Polymeric carbohydrates with ten or more monomeric units that are not hydrolyzed by the endogenous enzymes in the body’s small intestine are considered as dietary fiber [184]. The human body uses carbohydrates in D-glucose, while almost all of them are efficiently digested and absorbed into the body. When a carbohydrate-containing food is consumed, the glycaemic response occurs (a respective rise and subsequent decrease in blood glucose level) following the digestion and absorption rate and insulin’s action to normalize the blood glucose level [44].

The implementation of emerging technologies during processing can optimize carbohydrates’ modification and extraction and generate innovative food products with altered physicochemical and functional properties (e.g., modified starch). For instance, Benito-Román et al. [124] applied a pressurized (25 bar) hot fluid process to dehulled waxy barley for the recovery of β-glucans and phenolic compounds. As the temperature increased from 155 to 175 °C, a lower yield of β-glucan (but a higher yield of polyphenols) was obtained due to the degradation of β-glucan. Minjares-Fuentes et al. [122] utilized ultrasound technology to extract high yields of hemicelluloses and their constitutive fractions (mannans, xylans, and xyloglucans) from grape pomace using short extraction times (2.6 h), low KOH concentrations (0.4 M), and room temperature. Benito-Román et al. [123] studied the recovery of β-glucan from barley using ultrasound-assisted extraction and found that the yield depends on the amplitude (the higher intensity applying, the highest output achieved) and on the extraction time, e.g., the more extended treatment, the lower molecular weight of β-glucan molecules received. Ultrasounds (400 W) have also been used to modify tapioca starch and resulted in increased swelling power of starch, especially when applying higher amplitude or prolonged sonication time [127]. Han et al. [126] treated corn with PEF (strengths up to 50 kV/cm) to evaluate its effect on starch properties. They referred that the gelatinization temperature and viscosity decreased with the electric field increase strength. Mhemdi et al. [125] investigated the electroporation of sliced beetroots with PEF (5 kV–1 KA generator, 600 V/cm, 100 μs pulse duration, 10 ms treatment time at 10 °C) to improve sugar beet sucrose production. According to the results, the obtained juice showed a higher sucrose concentration, while the concentrate was more transparent and less colored than that conventionally extracted by diffusion. Ultrasounds, microwaves, accelerated solvent extraction, PEF, and ohmic heating have also been utilized during the recovery of inulin and oligofructose from different sources, resulting in increased yields, reduced processing times, solvent, and energy consumption [185].

### 5.3. Lipids

Fatty acids (such as omega-3, omega-6, and omega-9 fatty acids) and other minor lipid compounds (e.g., tocols, phytosterols, phospholipids, and glycolipids) have been shown to exhibit health-promoting properties by affecting the body’s physiological functions positively. The physical properties of fats are the consequence of the triacylglycerol composition that affects the stability, structure, and nature of ordered phases. Lipids are food components that are very susceptible to oxidation and subsequently is one of the primary reasons causing food deterioration during processing and storage [182]. Besides, lipids can be oxidized and degraded during their ingestion, digestion, and eventual adsorption by the intestinal lumen [186]. The effect of non-thermal processing technologies on lipids is of particular interest as many of them are advanced oxidation processes. Special attention is required in the control of processing parameters, including treatment time for HPP, Ultrasounds, PEF and cold plasma, temperature for HPP, PEF and ultrasounds, power and amplitude for ultrasounds, intensity and strength of electric field for PEF, and flow and frequency for cold plasma [182].

For example, according to Akram et al. [128], γ-irradiation in different meat products is followed by the development of off-flavors (e.g., 1-heptene and 1-nonene) mainly due to enhanced lipid oxidation. On the other hand, HPP has been suggested for fat reduction by 35% in dry-cured fermented sausages, enabling the stable incorporation of olive oil without affecting consumer acceptability [129]. Torkamani et al. [130] studied the effect of ultrasounds on lipids oxidation in Cheddar cheese whey, reporting the development of lipid oxidation volatile compounds. Besides, the application of cold plasma processing for the decontamination of walnuts and peanuts has resulted in a 20%-increase of lipids peroxide value at higher power and longer treatment time.

### 5.4. Essential Minerals

Minerals such as calcium, iron, and zinc have many body functions, and if not received in sufficient amounts, deficiencies are observed through specific and nonspecific symptoms. Minerals must be incorporated through the diet, but their content varies a lot despite chemical forms and quantity among the different foods and dietary habits. For instance, calcium is mainly received from dairy products (e.g., 98–1290 mg Ca/100 g cheese), but in many cases, it is not abundant in these products, e.g., only 13 mg/100 g butter. Iron content in meat ranges from 2 to 4 mg/100 g, while the highest concentration of iron can be found in legumes (7–10 mg/100 g) among the different vegetables. Besides, the primary sources of zinc are meat products (20–60 mg/kg in meat), seafood and fish (>15 mg/kg), and milk (3–5 mg/L in milk) [31]. Nevertheless, if the bioavailability is low, the amount received by consuming the above foods is not always adequate to reach dietary requirements. The consumption of fortified foods has boosted essential mineral uptake of individuals with respective deficiencies. During fortification of foods, the bioaccessibility of minerals should be evaluated, taking into account several parameters such as diet’s inhibitors and promoters, but also the determination methodologies is of critical importance.

There is very little information about the impact of non-thermal technologies on essential minerals. Similar to conventional processes, the non-thermal technologies do not affect minerals directly, but they generate changes in the physical properties and structure of the associated macromolecules. For example, it has been shown that the application of (450–650 MPa for 3 min at 20 °C) on soy and milk smoothies did not affect the content of minerals significantly (e.g., magnesium, manganese, zinc, calcium, sodium, iron, potassium, and copper), sugars and organic acids during treatment and storage [187]. Sila et al. [188] investigated the impact of HPP (200–500 MPa for 15 min, 20–60 °C) on carrots fortified with calcium. The latest practice is used to retard the rate of thermal softening during the processing of processed carrots. In another study, Speroni et al. [189] investigated the impact of HPP and calcium (2 to 25 mM) on soybean proteins’ gelation. For low calcium concentrations, HPP resulted in gels with low stiffness compared to control samples. For higher calcium concentrations, HPP of dispersions resulted in a decrease of gel stiffness in glycinin and the opposite in the presence of β-conglycinin. Dynamic, ultra-high pressure homogenization treatment (300 MPa) has also been reported to increase the viscosity of whey protein-depleted milk concentrates more intensively at low mineral contents (5% instead of 10% w/w) [190]. Similarly, Zamora et al. [134] referred that both ultra-high-pressure homogenization triggered changes in protein interactions (e.g.,) within rennet curds through ionic bonds with calcium salts. HPP (100–600 MPa) induced changes in the mineral balance of milk (e.g., solubilization of colloidal calcium phosphate) and subsequently increased solubilization of α1- and β-casein due to the disruption of hydrophobic interactions [132,133].

### 5.5. Vitamins

Vitamins are a variety of dietary compounds necessary for the correct maintenance of the body’s functions. There is an increased interest in the vitamin supply through the diet through minimally processed and nutritious foods and drinks [7]. Depending on their solubility, vitamins can be classified into soluble (e.g., vitamins C and B) and fat-soluble (Vitamins D, A, K, and E) groups [191]. The implementation of non-thermal technologies aims at improving the stability and bioaccessibility of vitamins in foods to maintain their functionality through their metabolism and health-promoting activity. Nevertheless, Vitamin C is highly modified after γ-irradiation depending on the food type. For instance, the irradiation of garlic and onion does not affect the content of Vitamin C [137,138]. In contrast, irradiated potatoes showed a loss in vitamin C content at the early stages of storage. Nevertheless, vitamin C was higher in irradiated samples than in untreated controls after many storage months [135,136]. Vitamin is highly reactive to irradiation, but if the treatment doses are low enough, the impacts on the organoleptic properties are minimized. On the other hand, Vitamins B, K, and D, as well as carotene, are moderately sensitive to irradiation [136].

Sancho et al. [139] investigated the impact of HPP on hydrosoluble vitamins (B1, B6, and C) and reported a significant reduction of Vitamin C, but no significant losses of vitamins B1 and B6 after the treatment. According to Polydera et al. [140], a short HPP treatment (500 MPa at 35 °C for 5 min) resulted in higher retention of ascorbic acid during post-processing storage of orange juice to the conventional thermal pasteurization. Besides, PEF treatment has also resulted in better preservation of orange juice’s Vitamin C amounts than heat pasteurization [141]. Similar results have also been reported by Elez-Martínez et al. [142] and Elez-Martínez and Martín-Belloso [143]. Finally, it has been referred that processing of juices with ultrasounds provides beneficial effects on the ascorbic acid content of an orange, guava, and Kasturi lime juice [144,145,146].

### 5.6. Polyphenols

Polyphenols are secondary metabolites found in all plants and contain one aromatic ring with one or more hydroxyl groups in their structure. They are classified into two main groups; flavonoids and non-flavonoids [27], while their average daily intake in the diet is approximately 1 g per person [192]. The bioavailability of polyphenols is generally low compared to other nutrients [193]. It varies greatly depending on the chemical structure of each polyphenol that affects the rate and extent of absorption and the form of metabolites present in human plasma [194]. Only the low molecular weight polyphenols (that account for approximately from 5 to 10%) can be absorbed in the small intestine [50], while the remained oligomeric and polymeric compounds reach the large intestine where bacteria act with their enzymes producing metabolites that exert an altered physiological activity compared to parent polyphenol [195]. The bioavailability of polyphenols can be improved by transforming their basic structure into an active form [196], e.g., forming inclusion complexes with cyclodextrins [197] or incorporating them into nanocarriers [198].

The different technologies applied during the production of foods can lead to changes in the content and bioactivity of polyphenols. Patras et al. [147] studied the effects of HPP on the antioxidant activity and content of polyphenols of blackberry and strawberry purées denoting HPP treatment preserved the color, anthocyanins content, and antioxidant activity of purées compared to conventional thermal treatment that showed a significant loss. Similarly, Ferrari et al. [148] that HPP treatment results in an enhanced extractability of anthocyanins in red fruit derivatives. Besides, the application of PEF (energy input of 10 KJ/Kg) for the recovery of bioactive compounds from grape pomace led to a higher yield of anthocyanin monoglucosides by 35% [149]. Donsì et al. [150] investigated a vinification process assisted by PEF treatments to increase the permeability of grape skins and subsequently boosting polyphenols concentration of red wines. According to their foundlings, PEF treatment resulted in a two-fold increase of polyphenols and a 30% increase of anthocyanins content, leading to improved color intensity and antioxidant activity of the produced wine. Besides, the application of ultrasounds has also been referred to increase (by 24%) the recovery of polyphenols from pomegranate peels compared to conventional liquid extraction [151].

### 5.7. Glucosinolates

Glucosinolates (GLs) and isothiocyanates (ICs) (their bioactive hydrolysis products) constitute a distinctive group of secondary metabolites (sulfur- and nitrogen-containing glycosides) found in the plant order Brassicales that includes different vegetable crops of the *Brassica* genus such as cauliflower, cabbage, rapeseed, kale turnip, horseradish, broccoli, Brussels sprouts, and mustard species. Various in vitro and in vivo studies have shown that GLs and some ICs provide beneficial health properties (e.g., antimicrobial, antidiabetic, anticancer, etc.) and play a significant role in plant physiology system and human health [73].

Different non-thermal technologies have been used to treat agricultural products containing GLs and ICs, mainly for recovery purposes. To this line, Ebrahimi et al. [152] applied microwave (800 W, 2–6 min) assisted extraction reporting a significant decrease (up to 59%) in GLs contents of canola seed compared to untreated samples, while degradation increased as long as irradiation time was extended. On the other hand, Omirou et al. [153] used microwaves to extract GLs from *Eruca sativa* seeds and found that it was more effective than conventional methods and ultrasounds. HPP of broccoli can cause the hydrolysis of GLs during treatment that leads to the recovery of ICs without affecting their beneficial properties [110]. Alvarez-Jubete et al. [154] studied the impact of HPP (200–600 MPa/20–40 °C) on white cabbage (*Brassica oleracea* L. var. capitata alba), referring to an increase in ICs content compared to blanched samples. In another approach, Kovacevic et al. [75] investigated the extraction of steviol glycosides (stevioside and rebaudioside A) and antioxidants compounds from Stevia using pressurized (10.34 MPa) hot water extraction, showing that the temperature was the most critical extraction parameter (highest yield at obtained at 160 °C) for extraction yields. In contrast, the highest recoveries of all bioactive compounds (except carotenoids) got at 160 °C. The extracts received at longer processing times contained more steviol glycosides, condensed tannins, and chlorophyll A. Finally, Frandsen et al. [155] denoted an increased extraction of glucobrassicin (6 μmol/g dry matter) and a decrease in aliphatic glucosinolate contents from broccoli puree using PEF treatment (electric field strength equal to 3 kV/cm). According to the authors, PEF increased the permeabilization of membranes that led to GLs degradation due to myrosinase catalyzed hydrolysis reactions.

### 5.8. Carotenoids

Carotenoids comprise a class of antioxidants and natural colorants widely distributed in nature, particularly in all colored flowers, vegetables, and fruits. These compounds have diverse roles in photochemistry, photobiology, nutrition, and medicine due to their health effects and the fact that they exert preventive activity against chronic diseases. Carotenoids’ efficiency depends on their bioaccessibility and bioactivity and their stability during extraction processes, handling, and storage [31]. Although few references denote a reduction in carotenoid content during processing, it is crucial to avoid isomerization and oxidation processes that show reversible effects on the carotenoids’ bioavailability. For instance, cis-lycopene demonstrates higher bioavailability than trans-lycopene because cis-isomers solubilize easily in bile acid micelles [199].

Food technologists have applied various emerging technologies to recover carotenoids from different natural sources and preserve their functionality. The carotenoid bioaccessibility can be increased by using high-pressure homogenization (from 40 to 400 MPa). For example, Xie et al. [157] have reported an increase in the bioaccessibility of lutein recovered from Desmodesmus sp. F51 (highly thermotolerant microalgae). Rodriguez-Roque et al. [158] investigated the bioaccessibility of carotenoids from fruit beverages treated with high-pressure homogenization and reported a 15% increase in carotenoids digestibility compared to the untreated sample. On the other hand, HPP (450Mpa and 600Mpa) has been applied to treat refrigerated papaya, melon, milk, and orange smoothies, showing a 25% increase of carotenoid content (lycopene, β- and α-carotene) [159]. HPP treatment of carrot juice accelerated the isomerization and oxidation of carotenoids, reducing their stability [160]. Besides, during the pasteurization of carrot juice with ultrasounds, the concentration of β-carotene increased up to 95 µg/g, accelerating a pro-oxidant effect that leads to an increase of the sample’s peroxide content [156].

### 5.9. Food Aroma Compounds

Aroma compounds are among the main components of food, affecting their sensory profile and, subsequently, consumers’ acceptance. They are compiled by low molecular weight (<400 Da), lipophilic organic molecules (volatile or not) that exist inherently in foods as a result of different enzymatic, fermentation, and physiological processes, or generated during their extraction or foods processing and storage. Being volatile, aroma compounds are easily lost during traditional thermal treatment of foods, and thus non-thermal technologies comprise an essential alternative for their preservation.

Among the different emerging technologies, supercritical fluids extraction has been widely used for the recovery of aromas from natural products [15] and other processes, such as decaffeination [200]. Da Porto et al. [161] applied supercritical fluids (10–14 MPa at 40 °C) to recover aromas from hemp inflorescences. They mentioned an increased solubility of hydrocarbon sesquiterpenes and oxygenated sesquiterpenes together with undesirable compounds like cuticle waxes. In a similar approach, Bogdanovic et al. [162] applied a two-step (at 10 and 30 MPa, respectively) supercritical fluid extraction method to recover different components from lemon balm. The first step allowed the recovery of essential oils, while the second increased the extraction of heavy alcohols and waxes (high molecular weight compounds). The implementation of temperatures higher than 25 °C allowed the extraction of phenolics such as thymol and eugenol. Tekin et al. [163] reported the recovery of essential oils from clove using ultrasound technology. Solvent-free microwave extraction represents an alternative technology for extracting volatile compounds from aromatic herbs with adequate efficiency. For example, Chen et al. [164] extracted essential oils from citrus peel using this technology, observing higher yields than conventional hydrodistillation, although at a thermal degradation observed at higher microwave temperature. Another method for efficiently extracting essential oils from aromatic plants is ohmic-assisted hydro-distillation [165]. Last but not least, encapsulation plays a vital role in the preservation of functionality of aromatic compounds. To this purpose, several techniques have been applied, e.g., the formulation of nanoemulsions. For example, Salvia-Trujillo et al. [166] investigated relevant formulations of essential oils (thyme, clove, lemongrass, tea tree, palmarosa, marjoram, geranium, rosewood, sage, and mint), pointing out the nanoemulsions exhibited an increased and faster microbial inactivation, which resulted from an immediate release of antimicrobial aromatic compounds.

### 5.10. Enzymes

Enzymes are natural catalysts comprised of long amino acid chains (proteins). They attach to a specific substrate site and work minimizing the energy requirement required to activate any reaction occurring inside living organisms. Enzymes can be recovered from different sources, such as animals, plants, and microorganisms. Their activity depends on the substrate’s concentration and other physical conditions such as pH and temperature [201]. When the enzyme’s protein is being damaged through permanent changes, its function is modified. Deactivation of enzymes is traditionally conducted using thermal treatments that cause undesirable sensory changes to foods. To this line, emerging technologies such as ohmic heating, radiofrequency, and microwave heating are alternative processes [202].

For instance, polyphenol oxidases (PPOs), which are enzymes causing browning of fruits and vegetables [203], have been inactivated by processing Tender coconut water with ohmic heating (80 °C for 3 min at 20 V/cm) [167]. Abedelmaksoud et al. [168] also confirmed that ohmic heating could deactivate PPOs of apple juice. Deactivation occurs by modifying the enzyme’s surface charge due to the ionization of solution constituents [204]. Deactivation of PPOs and other enzymes (e.g., polygalacturonase) can also be performed by applying HPP that cause structural and functional modifications of enzymes due to damage to weak hydrogen bonding [205,206,207,208,209]. Even low pressures can alter enzymes activity (e.g., <100 MPa) [210,211]. For example, Gui et al. [169] referred to a decline in apple juice PPO activity from 57 to 38% by amplifying the pressure from 8 to 30 MPa. Many enzymes such as pectin methylesterase, lipoxygenase are deactivated by applying the PEF method at the energy around energy 0.3 kJ/mL [170,171,172,212]. Ultrasounds have also been reported as an efficient enzyme deactivation method (e.g., PPO in pineapple pulp and cantaloupe melon juice without disturbing the quality of fruits and vegetables [173,174,175].

## 6. Conclusions

Following literature data, non-thermal technologies have been proved to be an efficient, fast, and reliable way to preserve food components’ bioavailability (e.g., the bioaccessibility of carotenoids), improve their functional properties (e.g., increase the gelling capacity of proteins) as well as to increase the corresponding recovery yields. However, the application of emerging may lead to the degradation of polymers (e.g., proteins and carbohydrates) and the oxidation of labile compounds (e.g., lipids or glucosinolates) if applied for extensive treatment time, high intensity or relatively high temperatures. Thereby, monitoring and optimization of operational parameters for each separately are critical. Although many of these technologies have today been industrially demonstrated, more efforts are needed in this direction. Modern practices and food processing trends have generated innovations in components incorporation, fortification of foods, and ultimately, how we consume foods. To this line, more thorough investigations regarding the effects of emerging technologies on foods for the exploration of different parameters (e.g., bioaccessibility characteristics and bioactivity of food compounds, nutritional value, applications, and shelf-life of foods, as well as their sensory aspects and consumer acceptance).

## Figures and Tables

**Table 1:** The effects of emerging technologies on different food component.

Components	Emerging Technologies	Food	Results	Reference
Proteins	Ohmic heating	Seafood and surimi	Minimize proteins degradation, forming more rigid and elastic gels	[103,104,105]
	High pressure processing	Surimi	Improvement of emulsifying properties	[100]
	High pressure processing	Fish, meat, egg and soy albumin	Improvement of gelling capacity and formation	[106,107,108]
	High pressure processing	Meat	Improvement of apparent digestibility, without affecting the biological value or protein efficiency ratio	[109]
	High pressure processing	Tomatoes, carrots and broccoli	Negligible loss of beneficial substances, higher glucose retardation index and water retention	[110]
	Pulsed electric field	Eggs	Negligible conformation, denaturation and precipitation	[111,112]
	Prolonged and high-intensity pulsed electric field	Eggs and milk	Partial modification of proteins	[113,114]
	High intensity ultrasound	Whey	Increase of charge, hydrophobicity, surface activity, emulsifying properties, solubility, foaming capacity and flexibility	[115,116]
	γ-Irradiation	Fish	Degradation of protein	[117,118]
	γ-Irradiation	Soy, eggs, and whey	Decrease in viscosity	[119,120,121]
Carbohydrates	Ultrasounds	Grape pomace	Increased extraction yield of hemicelluloses, mannans, xylans and xyloglucans at shorter time	[122]
	Ultrasounds	Barley	Increased ultrasound intensity resulted in highest recovery yield and smaller β-glucan molecules	[123]
	Pressurized extraction	Dehulled waxy barley	Lower yield of β-glucan in higher temperatures due to its fragmentation	[124]
	Pulsed electric field	Beet roots	Extraction of higher sucrose yield and more clarified concentrate	[125]
	Pulsed electric field	Corn	gelatinization temperature and viscosity of starch decreased	[126]
	Ultrasounds	Tapioca	Increased swelling power of starch	[127]
Lipids	γ-Irradiation	Meat products	Development of off-flavors due to lipids oxidation	[128]
	High pressure processing	Meat products	Fat reduction in dry-cured fermented sausages	[129]
	Ultrasounds	Cheddar cheese whey	Development of lipid oxidation volatile compounds	[130]
	Cold Plasma	Walnuts and peanuts	Increase in lipids’ peroxide value	[130]
Essential minerals	High pressure processing, high pressure homogenization	Soybean, smoothies, milk, carrots	Changes in minerals balance and solubilization of macromolecules (e.g., protein) associated with them	[131,132,133,134]
Vitamins	γ-irradiation	Potatoes	Reduction in Vitamin C	[135,136]
	γ-irradiation	Garlic and onion	No effect on Vitamin C content at early stages of storage	[137,138]
	High Pressure Processing	Egg yolk and strawberry *coulis*	A significant reduction of Vitamin C, but no significant losses of vitamins B1 and B6 after the treatment	[139]
	High Pressure Processing	Orange juice	Higher retention of Vitamin C compared to pasteurization	[140]
	Pulsed Electric Field	Orange juice	Higher retention of Vitamin C compared to pasteurization	[141,142,143]
	Ultrasounds	Orange, guava and kasturi lime juice	Higher retention of Vitamin C compared to pasteurization	[144,145,146]
Polyphenols	High Pressure Processing	Blackberry and strawberry purées	Preservation of the color, anthocyanins content and antioxidant activity of purées	[147]
	High Pressure Processing	Red fruit derivatives	Enhanced extraction of anthocyanins	[148]
	Pulsed Electric Field	Grape pomace, wine	Higher yield of anthocyanins and polyphenols monoglucosides	[149,150]
	Ultrasounds	Pomegranatre peels	Increase of polyphenols yield	[151]
Glucosinolates	Microwave	Canola seeds	Degradation of glucosinolates as a factor of irradiation time	[152]
	Microwave, ultrasound	*Eruca sativa* seeds	Increased recovery of glucosinolates when applying microwaves instead of ultrasound and conventional methods	[153]
	High Pressure Processing	Broccoli	Hydrolysis of glucosinolates and recovery of isothiocyanates	[110]
	High Pressure Processing	White cabbage	Increased content of isothiocyanates compared to blanching	[154]
	Pressurized hot water extraction	Stevia	Increased recovery of steviol glycosides, condensed tannins, and chlorophyll A	[75]
	Pulsed Electric Field	Broccoli	Increased yields of glucobrassicin and decreased yields in aliphatic glucosinolate contents	[155]
Carotenoids	Ultrasounds	Carrot juice	Increased content of β-carotene that led to a pro-oxidant effect	[156]
	High Pressure Homogenization	Microalgae	Increased bioaccessibility of lutein	[157]
	High Pressure Homogenization	Fruit beverages	Increased digestibility of carotenoids	[158]
	High Pressure Processing	Orange, papaya, melon and milk smoothies	Increase of carotenoids’ (lycopene, β- and α-carotene) content	[159]
	High Pressure Processing	Carrot juice	Increased isomerization and oxidation of carotenoids	[160]
Aroma compounds	Supercritical fluids extraction	Hemp inflorescences	Increased solubility of hydrocarbon sesquiterpenes and oxygenated sesquiterpenes together with undesirable cuticle waxes	[161]
	Supercritical fluids extraction	Lemon balm	Recovery of essential oils, heavy alcohols, waxes, thimol and eugenol	[162]
	Ultrasounds	Clove	Recovery of essential oils	[163]
	Solvent-free microwave	Aromatic herbs	Increased essential oils extraction, but degradation of them at higher temperatures	[164]
	Ohmic assisted hydro-distillation	Aromatic herbs	Increased extraction of essential oils	[165]
	Nanoemulsions	Thyme, clove, lemongrass, tea tree, palmarosa, marjoram, geranium, rosewood, sage and mint	Increased microbial inactivation due to the fast release of essential oils	[166]
Enzymes	Ohmic heat	Coconut water, apple juice	Deactivation of polyphenol oxidase	[167,168,169]
	Pulsed electric field	Orange juice, tomato juice, carrot	Deactivation of pectin methylesterase, lipoxygenase	[170,171,172]
	Ultrasounds	pineapple pulp and cantaloupe melon juice	Deactivation of polyphenol oxidase	[173,174,175]

## Data Availability

Not applicable.
